# Retained Wireless Capsule Endoscope in a Girl with suspected Crohn’s Disease

**DOI:** 10.21699/ajcr.v7i4.466

**Published:** 2016-09-01

**Authors:** Koushik Herle, Susan Jehangir

**Affiliations:** Department of Pediatric Surgery, Christian Medical College, Vellore, Tamil Nadu, INDIA

**Keywords:** Wireless capsule endoscopy, Retained capsule, Crohn's disease, Pediatric endoscopy

## Abstract

Wireless capsule endoscopy (WCE) is one of the great milestones in the field of gastroenterology. It is versatile in image acquisition, painless and can reach parts of the small bowel not amenable to conventional endoscopy. The commonest complication with WCE is retention of the capsule. We report a case of retained capsule in a child who was being investigated for obscure gastrointestinal bleeding (OGIB). Operative intervention was required for its retrieval after two weeks of expectant management.

## CASE REPORT

A 13-year-old girl underwent WCE for recurrent episodes of chronic abdominal pain and melena. Various investigations had been performed including workup for tuberculosis (TB), ultrasound abdomen, contrast meal and follow-through, upper and lower gastrointestinal tract (GIT) endoscopy, but none of these were diagnostic. A WCE (PillCam SB 3, Covidien) was performed using a capsule 11mm x 26mm with a battery life of >8hours. Ten hours after ingestion the capsule stopped at mid ileum. No significant lesion was seen. A computed tomogram showed the capsule in the ileum with multiple focal short segments of circumferential bowel wall thickening in the mid and distal ileum. Since the child was asymptomatic we decided to wait and watch. Two weeks later, she developed abdominal pain and multiple episodes of bilious vomiting. There was no abdominal distension or fever and her vital signs were within normal range. An erect abdominal x-ray showed the capsule in the small bowel loops with few air fluid levels (Fig. 1). A decision was taken to surgically extract the capsule. Intraoperatively, the capsule was found approximately 100cms proximal to ileocecal junction lying proximal to an area of luminal narrowing. The terminal ileum showed skip lesions with creeping fat. The capsule was extracted from an enterotomy at the site of stricture and a stricturoplasty was done. A biopsy was also taken from the stricture site. The patency of distal bowel lumen was confirmed. Postoperative recovery was uneventful. Histopathology showed non-specific inflammatory infiltrates, mild chronic active inflammation with no granulomas, malignancy or infective process. She is currently on mesalamine in view of intraoperative findings and is on regular follow up.

**Figure:**
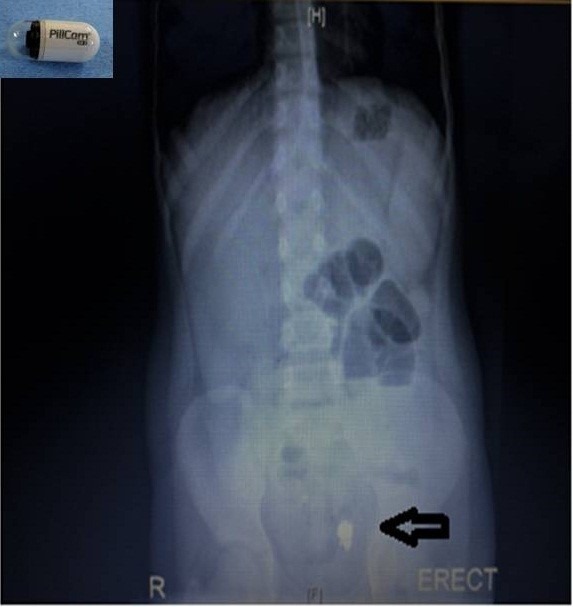
Figure 1:X-ray abdomen showing the retained capsule. Inset—PILLCAM^TM^ SB3 capsule system.

## DISCUSSION

The introduction of capsule endoscopy to the diagnostic armamentarium has positive yield for the diagnosis of inflammatory bowel disease (IBD).[1] The clinical application of WCE became clear in terms of pre-procedure preparation and investigations, indications, method of placement of capsule and limitations, with the consensus report from the Spanish society of gastroenterology, hepatology and nutrition published in 2015. [2] The American Society of Gastrointestinal Endoscopy guidelines state that WCE should be the first line investigation after a negative bidirectional endoscopy.[3]

WCE can get retained in the GIT which is defined as non-expulsion of the capsule in the bowel for more than two weeks. Capsule retention may cause obstructive symptoms or may remain completely asymptomatic.[4] The longest time for which a capsule has been left in situ is 38 months.[6] A retained capsule can be extracted surgically or endoscopically if accessible. The advantage of surgical extraction is the ability to treat the bowel stricture in addition to obtaining tissue biopsy. Cohen et al in a meta-analysis of 723 paediatric patients found retention rates of 2.6%.[7] Liao et al in their meta-analysis found that retention rate in case with suspicion of Crohn’s disease was 1.3% in contrast to established Crohn’s with a rate of 2.1%.[3] The problem of retention can be addressed by using patency capsule test. The capsule made of cellulose material is used before WCE and if that get retained then it may be a contraindication to WCE. Magnetic resonance enterogram (MRE) can also be performed before WCE to gain information about any stricture where a capsule can get stuck.[2] In conclusion, WCE is an important tool for identifying small bowel pathology. In suspected cases of GIT strictures, it is prudent to perform a patency capsule test or a MRE before performing WCE.

## Footnotes

**Source of Support:** Nil

**Conflict of Interest:** None declared

